# Affective and interpersonal expression in young adults during simulated interviews: implications for mental health research

**DOI:** 10.3389/fpsyt.2026.1946199

**Published:** 2026-10-28

**Authors:** Tianyi Zhang, Kaining Chang

**Affiliations:** Key Laboratory of Child Development and Learning Science (Ministry of Education), School of Biological Sciences and Medical Engineering, Southeast University, Nanjing, China

**Keywords:** affective communication, personality expression, simulated interview, social-evaluative interaction, young adult mental health

## Abstract

**Background:**

Simulated interviews could provide behavioral information for young-adult mental-health research, including studies of social-evaluative anxiety. Their value depends on reliable observer impressions and interpretable links to participants’ self-reports. We examined personality expression, affective communication, self-observer correspondence, and competence impressions in emerging adults.

**Methods:**

Two video-interview protocols were administered to participants aged 18–23 years: an occupationally framed protocol ( n=55) and a broader personality-oriented protocol ( n=33); 30 participants completed both. The protocols covered parallel HEXACO-related domains but differed in both framing and question content. Participants completed HEXACO self-reports and selected one of five valence-arousal categories after each response. Four observers rated personality expression, affective communication, and competence. We distinguished focused domain-matched tests from secondary question-level tests and exploratory correlation matrices, reported 95% confidence intervals, used permutation tests, and controlled the false-discovery rate within analysis families.

**Results:**

Self-reported HEXACO scores were similar across protocols except for lower Agreeableness in the broader protocol (mean difference =−0.23, 95% CI [−0.35,−0.12], q=0.004). Global valence was more positive in the broader protocol (mean difference =0.30, 95% CI [0.15,0.45], q<0.001), whereas the arousal difference was uncertain. Reliability was heterogeneous: personality and competence ICCs ranged from 0.27 to 0.65 and 0.35 to 0.64 in the occupational protocol and from 0.33 to 0.58 and 0.22 to 0.73 in the broader protocol. Question-level affect ICCs were approximately 0.00 to 0.60 and −0.91 to 0.03, respectively. Occupational self-observer correlations for Extraversion and Agreeableness survived correction; no broader-protocol personality correlation did. Strong same-observer personality-competence associations survived correction in both protocols, but broader-protocol affect-competence associations did not.

**Conclusions:**

Affective reports differed between protocols, but question content also differed, precluding a pure context effect. Observer-rating reliability and self-observer correspondence identify conditions for future mental-health studies pairing simulated interviews with validated symptom and functioning measures.

## 1 Introduction

Social connectedness refers to the experience of acceptance, belonging, support, and meaningful relationships rather than simply the number of social contacts a person has (1–3). It is a prominent developmental and public-health concern because social relationships are closely tied to well-being across adolescence and emerging adulthood (4, 5). Longitudinal and recent youth-mental-health work further identifies school belonging and social connection as important psychosocial targets (6, 7). This literature provides a broad motivation for studying how young people present themselves and are perceived during consequential interactions.

Most connectedness research relies on self-reports of belonging, loneliness, support, or relationship quality (2, 3). Such measures appropriately capture subjective experience, but they answer a different question from observer ratings of a person’s behavior in a specific interaction. A participant can experience acceptance or isolation without displaying a unique behavioral signature, and an observer can form an impression that does not match the participant’s broader self-view.

A specific measurement question in adolescent and young adult psychiatry is whether observers can reproducibly rate young adults’ affective communication and personality expression during brief social-evaluative interactions, and how those ratings relate to participants’ self-reports. Establishing these properties matters before behavioral observations are paired with validated symptom and functioning measures in studies of social-evaluative anxiety or adjustment during the education-to-work transition (8, 9).

### 1.1 From trait self-perception to situation-specific expression

The HEXACO model organizes personality into Honesty-Humility, Emotionality, Extraversion, Agreeableness, Conscientiousness, and Openness (10). These domains distinguish ethically oriented interpersonal behavior, emotional sensitivity, social activation, cooperation, task regulation, and openness to ideas and experiences. Personality self-reports summarize how individuals typically understand themselves across situations. During emerging adulthood, those trait self-views coexist with continuing development in narrative identity and the ways people organize personal experiences when describing themselves to others (11). An open-ended interview response is therefore not a second administration of a trait questionnaire. It is a bounded opportunity for a participant to select, narrate, and express trait-relevant behavior.

Person-situation perspectives emphasize that behavioral expression depends jointly on personal characteristics and the features of the eliciting situation (12). A person can report a relatively stable trait while displaying different aspects of that trait when asked about cooperation, setbacks, ethical choices, or unfamiliar experiences. A related distinction appears in emotion-regulation research, which separates trait-like regulation tendencies from situation-dependent regulatory ability (13). In the present study, this distinction motivates attention to protocol and question content, but it does not license a causal interpretation: the occupational and broader questions were not identical stimuli with only one contextual feature manipulated.

Self-reports and observer reports also have different informational advantages. Self-reports provide access to internal motives, feelings, and behavior across many settings; observers have access to visible and audible behavior in the sampled interaction (14, 15). The Realistic Accuracy Model and zero-acquaintance research suggest that accurate judgment depends on whether trait-relevant cues are available and used by the observer (16, 17). The Self-Other Knowledge Asymmetry model likewise predicts that highly visible traits can be easier for observers to judge than traits grounded in private motives or internal experience (18). Agreement should consequently vary across traits and tasks rather than being uniformly high or low.

Discrepancies between self-, meta-, and peer evaluations have also been documented in research on loneliness and social skills (19), while agreement in personality assessment varies with observers’ relationship to the target (20). These findings make self-observer correspondence theoretically informative, but they also set an important boundary. In the present zero-acquaintance setting, low correspondence cannot by itself be labeled poor self-knowledge, failed communication, loneliness, or impaired social functioning. Those interpretations would require direct measures and a design capable of separating the participant’s self-knowledge from cue expression, cue availability, and observer use.

### 1.2 Simulated interviews as social-evaluative contexts

Simulated interviews provide a standardized setting in which participants recall experiences, organize narratives, and respond while being evaluated. Structured and asynchronous video-interview research shows that such tasks can elicit information about past behavior, personality expression, and interview performance (21–23). Open-ended assessments can complement fixed-response questionnaires because they require participants to generate behavioral examples and construct a spoken account rather than endorse predefined statements (24, 25). For emerging adults approaching the university-to-work transition, this format is also ecologically relevant to increasingly common digital selection and training environments.

The simulated interview is used here not as an alternative to a qualitative research interview, but as a standardized behavioral-elicitation and observer-rating paradigm. All participants answered the same questions under the same recording conditions, which preserved comparability while allowing relatively spontaneous speech, vocal delivery, facial behavior, and visible engagement to enter the behavioral sample. This combination makes it possible to examine self-perception, expressed behavior, and observer judgment within the same event. Its ecological relevance is specific to selection-like asynchronous or one-way video settings, in which brief recorded answers are evaluated later. It does not imply equivalence to a live job interview, an actual hiring decision, a qualitative research interview, or everyday social interaction.

At the same time, a simulated interview is not equivalent to a natural relationship. Communication is largely one-directional, the questions constrain what can be expressed, and participants can manage the impressions they present. Employment interviews can serve multiple goals, and impression-management behavior has been observed in asynchronous video interviews across countries (26, 27). Observer ratings from this setting should therefore be described as impressions based on a limited behavioral sample rather than as direct assessments of enduring personality or everyday social functioning. Behaviorally anchored ratings can make the coding task more explicit (28), but they do not remove the sampling limits of the interview itself.

Question design determines which personal experiences and behaviors become available to observers. Occupational questions tend to emphasize role performance, responsibility, cooperation, decision making, and competence, whereas broader questions can invite more varied personal or emotional narratives (24, 29). Digital job interviews, virtual-reality interview simulators, and avatar-based interview training have also been used to study stress, behavior, feedback, or anxiety in evaluative settings (8, 30, 31). More generally, social-evaluative threat can operate through concerns about how the individual, relational, or collective self will be judged, and affective reactivity can differ between everyday interpersonal stress and acute evaluation (9, 32). These literatures support treating interview responses as task-sensitive, but they do not show that the two protocols used here differ solely in “evaluative pressure.

In this study, both protocols comprised six questions aligned broadly with the HEXACO domains, yet the paired questions were not word-for-word equivalents. They differed simultaneously in topic, wording, requested autobiographical content, emotional salience, and cognitive demands, in addition to occupational framing. The occupational and broader protocols also involved partly overlapping samples rather than a fully counterbalanced within-person experiment. Accordingly, comparisons between them are descriptive protocol differences. Any explanation in terms of evaluation, self-presentation, or narrative affordances remains a hypothesis for future controlled work.

### 1.3 Personality and affect as interpersonal signals

Personality and affect provide related but non-equivalent information in social interaction. Personality traits describe broad patterns of behavior and experience, whereas affect captures a more immediate state. Work on personality and emotions in social interactions emphasizes that both the person’s characteristics and the unfolding interaction contribute to what is expressed and perceived (33). Emotions also carry social-signal value because observers use affective displays to infer intentions, appraisals, and likely actions (34, 35). In mediated communication, emotional tone can influence engagement and peer acknowledgement, although evidence from those settings should not be treated as direct evidence about interview performance (36).

The distinction between experience and expression is central to the present measures. Participants reported their affect using valence and arousal categories derived from the circumplex model (37); observers separately rated the affect communicated in the recorded response. Measures of emotion do not necessarily converge because subjective experience, expressive behavior, and observer inference capture different components of an emotional episode (38). Emotion suppression and expression can also have different interpersonal correlates (39). Thus, self-observer affect correspondence indicates whether reported experience was legible in the available audiovisual behavior, not whether either reporter had privileged access to a single error-free emotional score.

Interview research further illustrates why affective cues require cautious interpretation. Enthusiasm intensity can be evaluated differently depending on how it is expressed, and anxious nonverbal behavior can influence interview-performance ratings (40, 41). The same level of arousal can be perceived as energetic engagement, tension, or poor composure depending on accompanying verbal and nonverbal cues. Observer agreement is therefore an empirical prerequisite: if raters do not reliably perceive the same affective signal, correlations involving the aggregated observer score can be attenuated or unstable. Research linking emotional self-knowledge profiles to mental-health indicators underscores the possible future relevance of self-perception (42), but the present design neither measures psychiatric symptoms nor tests emotional self-knowledge as a clinical construct.

### 1.4 Observer impressions of competence

Observers can integrate personality-relevant and affective cues into broader judgments of competence. We organized competence impressions using the Great Eight taxonomy (43). The HEXACO framework provides theoretically plausible links between selected trait expressions and some of these dimensions. For example, Conscientiousness can be relevant to organizing/executing, and Extraversion to interacting/presenting. Prior work also relates HEXACO self- and observer reports to integrity-related criteria (44). These links motivate focused association tests rather than a claim that every trait should predict every competence dimension.

Crucially, competence in this study is an observer impression, not an independently verified performance outcome. The same observers rated personality expression, affective communication, and competence from the same video responses. Associations among those ratings can reflect coherent impressions, but they can also be strengthened by overlapping behavioral anchors, shared method variance, or halo effects. We therefore use the term *competence impressions* and interpret these associations as same-observer covariance. Independent assessors, objective performance criteria, or longitudinal outcomes would be required to establish whether the impressions predict competence beyond the rating context.

### 1.5 The Present Study

Relevant work on structured and asynchronous interviews, zero-acquaintance personality judgment, affective experience and perception, and competence evaluation has generally examined these measurement levels separately. The specific gap addressed here is the limited reliability-aware evidence on how trait self-reports, immediate affective self-reports, observer-rated personality and affect, and competence impressions correspond within the same brief audiovisual event. We therefore examine three linked relations: trait self-report with situation-specific personality expression, experienced affect with perceived affect, and observer-rated personality and affect with competence impressions. Observer agreement is evaluated as a prerequisite for interpreting each observer-based association, and the patterns are described across occupational and broader protocols.

The present exploratory study examined personality self-reports, affective self-reports, observer-rated expression, and competence impressions across two simulated interview protocols in emerging adults aged 18–23 years. Its contribution is methodological and descriptive: it characterizes what appears stable across the available protocols, which observer ratings are reliable, how self-reports correspond to observer impressions, and how different judgments made by the same observers covary. Within young-adult mental-health research, these findings distinguish observer ratings with preliminary reliability and correspondence from affect ratings requiring improved coding before links to symptoms or functioning can be tested. They do not validate a social-connectedness profile, a clinical marker, or an index of psychiatric vulnerability.

The occupational protocol included 55 participants, the broader protocol included 33, and 30 participants completed both. Because the protocols used non-equivalent questions and only partly overlapping samples, within-person and between-sample analyses are reported separately where appropriate. We addressed three research questions:

Research Question 1: How do self-reported HEXACO scores and affect differ between the occupational and broader interview protocols?

Research Question 2: Within each protocol, how strongly do trait self-reports and affective self-reports correspond to observers’ ratings of situation-specific personality expression and affective communication?

Research Question 3: Within the same observer-rating method, how are rated personality expression and affective communication associated with competence impressions?

For each question, we distinguish focused domain-matched tests from secondary question-level tests and broader exploratory matrices. We report effect estimates with uncertainty, apply multiplicity control to defined test families, and interpret patterns in light of observer reliability. This hierarchy is intended to prevent isolated, sample-specific correlations from carrying more evidential weight than the design supports.

## 2 Materials and methods

### 2.1 Research design

We conducted two study-specific video-interview protocols using similar procedures. Participants first provided demographic information and then recorded a 1-2-minute spoken response to each of six questions. Immediately after each response, they selected one of five affect categories based on the valence-arousal circumplex (37): pleasant/high arousal (+1,+1), pleasant/low arousal (+1,−1), unpleasant/high arousal (−1,+1), unpleasant/low arousal (−1,−1), or neutral (0,0). The first coordinate was analyzed as valence and the second as arousal. After the interview, participants completed the HEXACO-100 questionnaire (10).

The post-response valence-arousal choice asked participants how they themselves felt while producing the immediately preceding answer. It did not ask them to rate the pleasantness of the question, the quality of their answer, or their suitability for employment. The response-contingent timing anchored the self-report to a specific behavioral episode rather than to a retrospective summary of the entire interview. These reports served both to compare experienced affect across protocols and to examine its correspondence with observers’ affective impressions of the same recordings. Self-reported experience and observer-perceived expression were therefore treated as related but non-equivalent measures.

The six questions were used as a study-specific elicitation set organized around the six HEXACO domains. For each domain, one question placed the participant in an occupational scenario and the other elicited a broadly analogous personal or everyday experience. The occupational-question development followed the structured, past-behavior procedure documented for the AVI-6 database collection protocol. Candidate occupational questions were written to elicit concrete behavior relevant to central facets of the target HEXACO domains rather than generic self-description. Four personality experts reviewed the candidates independently, selected the most suitable question for each targeted domain, resolved disagreements through discussion until consensus, and then refined the wording. This development account applies to the occupational questions. The present paper did not develop or validate generic questions, and the broader comparison questions are not presented as products of the generic-question development procedure described in the AVI-6 records. The occupational questions emphasized professional accountability, collaboration, setbacks, task execution, innovation, and continued cooperation. The broader questions addressed related themes without a specific work role. **Table 1** documents the construct mapping and content differences. The questions were not a validated diagnostic interview and were not intended to be content-identical (10, 22, 29).

**Table 1:** Correspondence between the six questions in the occupational and broader personality interview protocols.

Question index	Occupational questions	Broader questions	Main contextual difference
Q1	A conflict between personal values and tangible benefits in a work setting.	A situation in which personal principles were challenged by an opportunity for individual gain.	**Occupational:** Integrity is examined within professional accountability.
			**Broader:** The question focuses on personal moral choice.
Q2	Initiating interaction and leading discussion in a newly formed team.	Establishing contact or creating an active atmosphere among unfamiliar people.	**Occupational:** Social initiative is examined through a leading role in a work team.
			**Broader:** The question focuses on social engagement in an unfamiliar environment.
Q3	Responding to a major project failure and communicating the setback to relevant people.	Describing the emotional response to failure and the use of social support.	**Occupational:** The question emphasizes responsibility after project failure.
			**Broader:** Greater attention is given to subjective emotional experience.
Q4	Planning a project and monitoring its implementation to maintain work quality.	Completing a time-limited task with attention to progress and detail.	**Occupational:** Conscientious behavior is examined through project responsibility.
			**Broader:** The question focuses on personal habits during task completion.
Q5	Developing a new solution to improve an existing work process.	Trying an unfamiliar method or exploring a non-traditional idea.	**Occupational:** Openness is expressed through practical workplace innovation.
			**Broader:** The question focuses on curiosity toward novel ideas.
Q6	Maintaining effective cooperation with a difficult colleague.	Responding to persistent disagreement or interpersonal conflict.	**Occupational:** The question emphasizes continued collaboration within a work relationship.
			**Broader:** The focus is on general responses to interpersonal conflict.

The occupational protocol therefore served as the study’s selection-like application context, whereas the broader protocol sampled related behavior outside a narrowly occupational role. The broader protocol was not treated as a generic qualitative interview or a perfectly content-matched neutral control. Competence ratings in the occupational protocol indexed observers’ impressions within this task, not hiring suitability, future job performance, or employability.

Accordingly, the comparison is between two complete protocols that differ in both framing and item content. Any observed difference can reflect wording, topic, emotional salience, cognitive demand, or role expectations. It is not a pure causal estimate of an occupational-versus-general context effect.

### 2.2 Participants

Data were collected between February and June 2026. Analyses were restricted to participants aged 18–23 years, whom we describe as emerging adults or young adults rather than adolescents. As shown in **Table 2**, the occupational protocol included 55 participants ( $M_{age}=21.09$, SD=1.57), the broader protocol included 33 ( $M_{age}=21.18$, SD=1.38), and 30 completed both protocols ( $M_{age}=21.10$, SD=1.42). The occupational sample included 26 men and 29 women; the broader sample included 18 men and 15 women; and the matched subgroup included 15 men and 15 women. Participants were undergraduate (94.3%) or master’s students (5.7%). This non-clinical university-based convenience sample is relevant to socially evaluative interactions during the transition toward employment, but it does not represent clinical or at-risk populations.

**Table 2:** Participant characteristics.

Experiment/subgroup	*n*​	Age, M (SD)	Gender	Educational background
Occupational	55	21.09 (1.57)	Male: 26 (47.3%)	University: 52 (94.5%)
			Female: 29 (52.7%)	Master’s: 3 (5.5%)
Broader	33	21.18 (1.38)	Male: 18 (54.5%)	University: 31 (93.9%)
			Female: 15 (45.5%)	Master’s: 2 (6.1%)
Matched subgroup	30	21.10 (1.42)	Male: 15 (50.0%)	University: 29 (96.7%)
			Female: 15 (50.0%)	Master’s: 1 (3.3%)

### 2.3 Observer reports

Four observers reviewed the video responses and rated personality expression, affective communication, and job-related competence. The available study records document a 9-hour training procedure for the personality-rating component (22). Before the first session, observers read background material and definitions for the HEXACO model and completed a HEXACO-adjective self-check that provided automated feedback. Common errors were discussed during training. The training session reviewed the rating framework and was followed by the Dispositional Insight Test, again with feedback. Observers then independently rated videos from 4–10 practice participants. At a follow-up calibration meeting, discrepant ratings were discussed so that the observers shared the same interpretation of the domain and facet definitions and applied common rating criteria. The records do not document a numerical pre-coding reliability threshold, a separate training curriculum for affective communication or competence, or formal blinding to participants’ self-reports and the study hypotheses. We therefore do not claim these procedures. The available rating matrices contained a score from every observer for every included participant. Response-level or participant-level ratings were averaged across the four observers for the main analyses.

#### 2.3.1 Personality expression

Observers rated the extent to which the participant expressed each HEXACO domain in the recorded responses. These situation-specific ratings are referred to as personality expression and are not treated as parallel trait questionnaires. Behaviorally anchored rating scales (BARS) described observable indicators at different levels (28). Ratings used a 5-point scale (1 = very low, 5 = very high) and allowed one decimal place.

#### 2.3.2 Affective communication

Observers coded perceived affective communication for each response using the same valence-arousal coordinate system. We retained question-level valence and arousal scores and averaged them across the six questions to obtain global observer-rated valence and arousal. These ratings reflect perceived expression, not direct access to participants’ experienced affect.

#### 2.3.3 Observer-rated job-related competences

The eight competence dimensions were drawn from the Great Eight framework (43). BARS translated these broad dimensions into behavioral anchors suitable for structured interview judgment (21). Ratings used the same 1–5 scale with one decimal place. After evaluating reliability, we calculated a professional total across all eight dimensions and a social-professional composite across supporting/cooperating, interacting/presenting, and adapting/coping. These outcomes are competence impressions formed by the observers, not independently verified performance.

### 2.4 Statistical analysis

Analyses were organized in three tiers. Focused analyses directly addressed the research questions using domain-matched variables: six HEXACO factors and global valence/arousal for RQ1 and RQ2. Six theory-guided personality-competence pairs and four global affect-competence associations for RQ3. Question-level affect analyses were secondary. The complete personality-affect-competence correlation matrices were exploratory and were used to show the broader pattern rather than to support individual inferential claims.

We evaluated inter-rater reliability before interpreting observer-based associations. ICC(1,k) was calculated separately for each observer-rated HEXACO factor, competence dimension, and question-level valence and arousal score. For the eight competence dimensions, we also calculated Cronbach’s alpha and the average inter-dimension correlation before constructing the professional total and social-professional composite. Poor affect reliability was treated as a limit on substantive interpretation rather than as a result that could be repaired statistically.

For RQ1, paired comparisons used the 30 participants who completed both protocols. We report the mean difference (broader minus occupational), its 95% confidence interval, the paired standardized effect size $d_{z}$, a two-sided sign-flip permutation p-value, and the cross-protocol correlation. These estimates describe protocol differences. They do not isolate a causal effect of contextual framing.

For RQ2, Pearson correlations linked each trait self-report to the corresponding mean observer expression rating and linked self-reported valence/arousal to observer-rated valence/arousal. We report Fisher-transformed 95% confidence intervals and two-sided permutation p-values. Spearman correlations were retained as rank-based sensitivity analyses. Because trait self-reports and brief behavior ratings differ in scope, correlations were interpreted as cross-method correspondence rather than measurement interchangeability.

For RQ3, the focused pairs were Extraversion with interacting/presenting, Agreeableness with supporting/cooperating, Openness with creating/conceptualizing, Conscientiousness with organizing/executing, Honesty-Humility with leading/deciding, and Emotionality with adapting/coping (43, 44). Global observer-rated valence and arousal were also correlated with the professional total and social-professional composite. These associations were interpreted as coherence within the same observer method, not prediction of independent competence.

To address multiplicity, Benjamini-Hochberg false-discovery-rate correction was applied within each stated family and protocol: RQ1 personality, RQ1 global affect, RQ1 question-level affect; RQ2 personality, RQ2 global affect, RQ2 question-level affect; RQ3 targeted personality-competence pairs, and RQ3 global affect-competence pairs. We report both permutation p-values and adjusted q-values, with conclusions based on estimates, 95% confidence intervals, reliability, and q-values rather than isolated unadjusted significance. **Supplementary Table S1** reports all focused and secondary results. The study was not preregistered, no *a priori* power analysis was conducted before data collection, and no prospective sampling stopping rule was documented. We therefore do not retrospectively label the focused analyses as confirmatory. The study remains exploratory and no causal, diagnostic, or psychiatric-risk inference is made.

We conducted a sensitivity power analysis based on the realized sample sizes to describe the effect sizes the study could detect, rather than presenting this post-collection calculation as an *a priori* analysis. For two-sided tests at α=0.05, the paired sample ( n=30) provided 80% power to detect $|d_{z}|\geq 0.53$ and 90% power for $|d_{z}|\geq 0.61$. For correlations, the occupational sample ( n=55) provided 80% power for |r|≥0.37 and 90% power for |r|≥0.42, whereas the broader sample ( n=33) provided 80% power for |r|≥0.47 and 90% power for |r|≥0.53. The paired-test values were obtained from the noncentral t distribution and the correlation values from the Fisher- z approximation. Because the Benjamini-Hochberg rejection threshold depends on the full set of observed p-values, there is no single fixed FDR-adjusted power value. As a conservative six-test benchmark using Bonferroni α=0.05/6, the 80%-power thresholds were $|d_{z}|\geq 0.68$, |r|≥0.45 for n=55, and |r|≥0.56 for n=33. Thus, the available samples were primarily sensitive to medium-to-large effects, and smaller associations can have been missed.

## 3 Results

### 3.1 Measurement properties and descriptive structure

As shown in **Table 3**, reliability varied substantially by construct and protocol. For personality expression, ICC(1,k) ranged from 0.27 (Emotionality) to 0.65 (Agreeableness) in the occupational protocol and from 0.33 (Emotionality) to 0.58 (Extraversion) in the broader protocol. For competence impressions, the ranges were 0.35 (organizing/executing) to 0.64 (analyzing/interpreting) and 0.22 (supporting/cooperating) to 0.73 (analyzing/interpreting), respectively. These values support averaging some dimensions but do not justify a general statement that all observer ratings were reliable.

**Table 3:** Observer-report reliability and competence internal structure.

Protocol	Analysis	n​	Estimate	Detail
Occupational	HEXACO observer reliability	55	ICC(1,*k*)=0.27-0.65	Lowest: Emotionality, highest: Agreeableness
	Competence observer reliability	55	ICC(1,*k*)=0.35-0.64	Lowest: organizing/executing, highest: analyzing/interpreting
	Affect observer reliability	55	ICC(1,*k*) ≈0.00-0.60	Question-level valence and arousal
	Competence internal structure	55	α=0.92​	Mean inter-dimension r=0.60, range: 0.36-0.75
Broader	HEXACO observer reliability	33	ICC(1,*k*)=0.33-0.58	Lowest: Emotionality, highest: Extraversion
	Competence observer reliability	33	ICC(1,*k*)=0.22-0.73	Lowest: supporting/cooperating, highest: analyzing/interpreting
	Affect observer reliability	33	ICC(1,*k*) =−0.91-0.03	Question-level valence and arousal
	Competence internal structure	33	α=0.93​	Mean inter-dimension r=0.61, range: 0.34-0.78

Affective communication ratings were markedly less reliable. Question-level valence and arousal ICC(1,k) values ranged from approximately 0.00 to 0.60 in the occupational protocol and from −0.91 to 0.03 in the broader protocol. Negative ICC estimates indicate that between-participant variance was not reliably larger than residual rater variance under the model; they do not indicate inverse agreement. We therefore treat broader-protocol observer-affect associations as descriptive and do not draw substantive conclusions from them. The eight competence dimensions had high internal consistency in the occupational ( α=0.92, mean inter-dimension r=0.60) and broader ( α=0.93, mean inter-dimension r=0.61) protocols, supporting the specified summary indices as measures of observer impression coherence.

### 3.2 Protocol differences in personality self-reports and affect

**Figure 1** summarizes paired protocol differences, with positive values indicating higher scores in the broader protocol. Five HEXACO mean differences were small (from −0.04 to 0.06), and their 95% confidence intervals included zero. Cross-protocol correlations ranged from r=0.66 for Openness to r=0.84 for Conscientiousness, indicating substantial rank-order consistency in self-reports across the two administrations.

**Figure 1:**
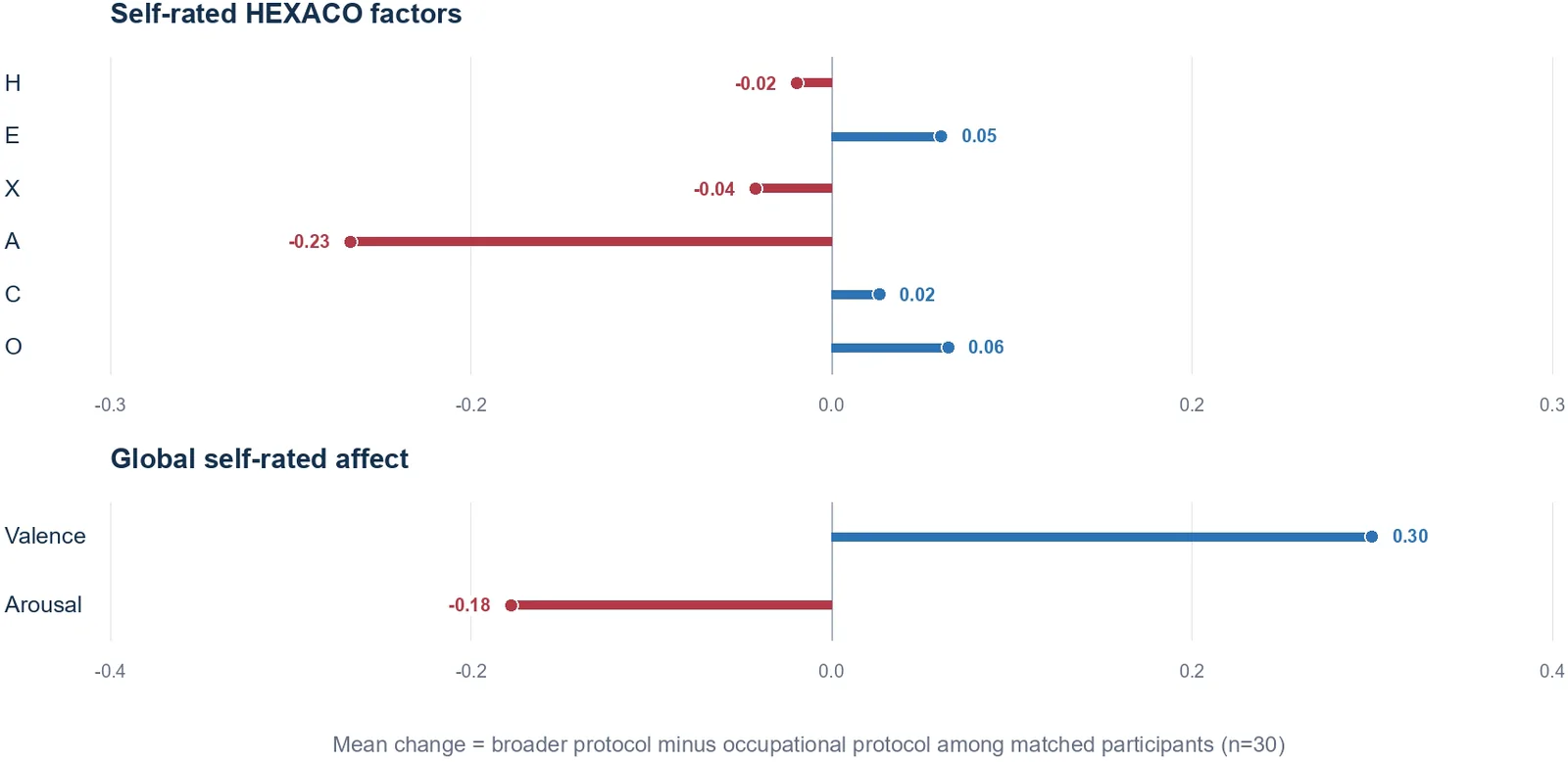
Protocol differences in self-rated personality and affect. Values represent the mean change from the occupational to the broader protocol among 30 matched participants. Confidence intervals and multiplicity-adjusted results are reported in **Supplementary Table S1**.

Agreeableness was lower in the broader protocol (occupational M=3.40, broader M=3.16, mean difference =−0.23, 95% CI [−0.35,−0.12], $d_{z}=-0.76$, permutation p<0.001, q=0.004), while its cross-protocol correlation remained high ( r=0.82). The result is a protocol-associated mean difference, not evidence that framing alone caused a change in trait Agreeableness.

Global self-rated valence was more positive in the broader protocol (occupational M=0.04, broader M=0.34, mean difference =0.30, 95% CI [0.15,0.45], $d_{z}=0.76$, p<0.001, q<0.001). Global arousal was lower on average, but the interval included zero (occupational M=0.18, broader M=0.00, mean difference =−0.18, 95% CI [−0.38,0.02], $d_{z}=-0.33$, p=0.089, q=0.089). Cross-protocol correlations were r=0.57 for valence and r=0.34 for arousal.

Secondary question-level analyses located the clearest differences in the Honesty-Humility question for valence (mean difference =0.63, q=0.029) and arousal (mean difference =−0.57, q=0.040), and in the Emotionality question for valence (mean difference =0.57, q=0.029). The remaining nine question-level comparisons did not survive correction (**Supplementary Table S1**). Because paired questions differed in content, these findings are descriptive protocol contrasts.

In summary, trait self-reports were similar across protocols except for Agreeableness, whereas global valence and selected question-level affect ratings differed. The data support protocol sensitivity of reported affect, but the design cannot attribute that sensitivity specifically to occupational framing.

### 3.3 Self-observer correspondence in personality and affect

**Figure 2** compares trait self-reports with mean ratings of situation-specific expression. In the occupational protocol, correspondence was clearest for Extraversion ( r=0.41, 95% CI [0.16,0.61], p=0.002, q=0.010) and Agreeableness ( r=0.33, 95% CI [0.08,0.55], p=0.011, q=0.033). The other four factor correlations did not survive correction.

**Figure 2:**
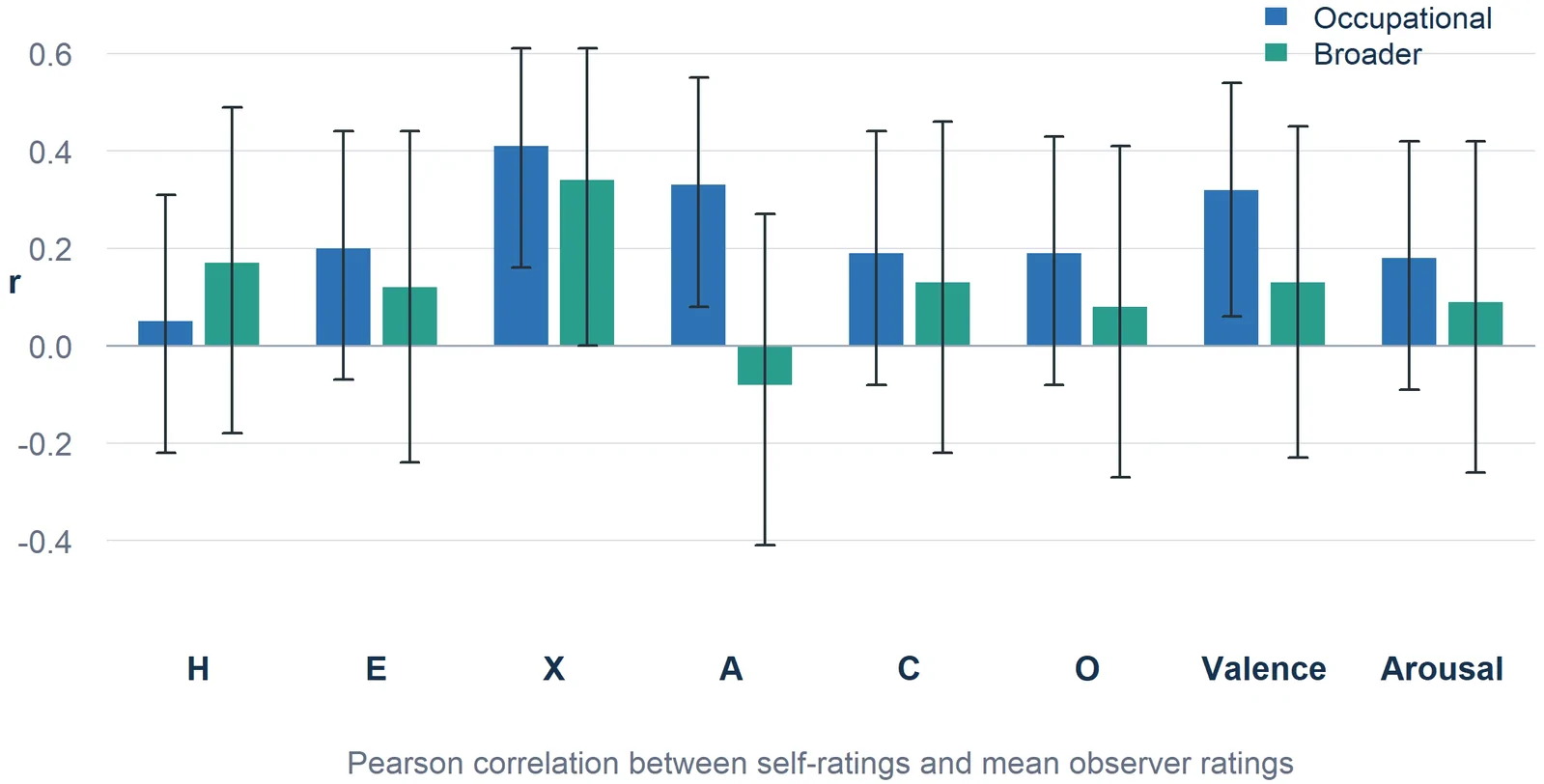
Self-observer correspondence in HEXACO factors and global affective states. Bars show Pearson correlations between self-reports and mean observer ratings; whiskers show 95% confidence intervals calculated using Fisher’s z transformation (occupational n = 55; broader n = 33). H, Honesty-Humility; E, Emotionality; X, Extraversion; A, Agreeableness; C, Conscientiousness; O, Openness. Exact estimates and adjusted q-values are reported in **Supplementary Table S1**.

These correlations do not show that the other traits failed to become socially interpretable. The self-report instrument describes typical traits across situations, whereas the observer measure samples behavior expressed during a brief structured task. Differences in scope, opportunity for expression, and rater reliability all constrain the maximum correspondence.

In the broader protocol, Extraversion had the largest estimate ( r=0.34, 95% CI [0.00,0.61], permutation p=0.050), but it did not survive correction across the six factors ( q=0.301); none of the broader-protocol personality correlations did. Spearman sensitivity analyses produced the same rank ordering, with occupational Extraversion and Agreeableness larger than most other pairs, but did not change the multiplicity-adjusted interpretation.

The intervals, particularly in the smaller broader-protocol sample, are wide; apparent differences in bar heights should not be read as direct between-protocol tests.

For global affect, occupational valence correspondence was r=0.32 (95% CI [0.06,0.54], p=0.019, q=0.038), whereas occupational arousal was uncertain ( r=0.18, 95% CI [−0.09,0.42], q=0.194). Broader-protocol valence ( r=0.13, q=0.637) and arousal ( r=0.09, q=0.637) were weak and, given the very low inter-rater reliability of observer affect in this protocol, are not interpreted substantively. None of the 24 question-level self-observer affect correlations survived correction within protocol (**Supplementary Table S1**).

**Figure 3** shows why caution is necessary. Occupational question-level affect ICCs ranged from approximately 0.00 to 0.60. Broader-protocol ICCs ranged from −0.91 to 0.03, showing that the four observers did not produce stable rankings of participants’ affective communication. We retain these values for transparency but do not use the broader-protocol affect correlations as evidence for interpersonal affect recognition.

**Figure 3:**
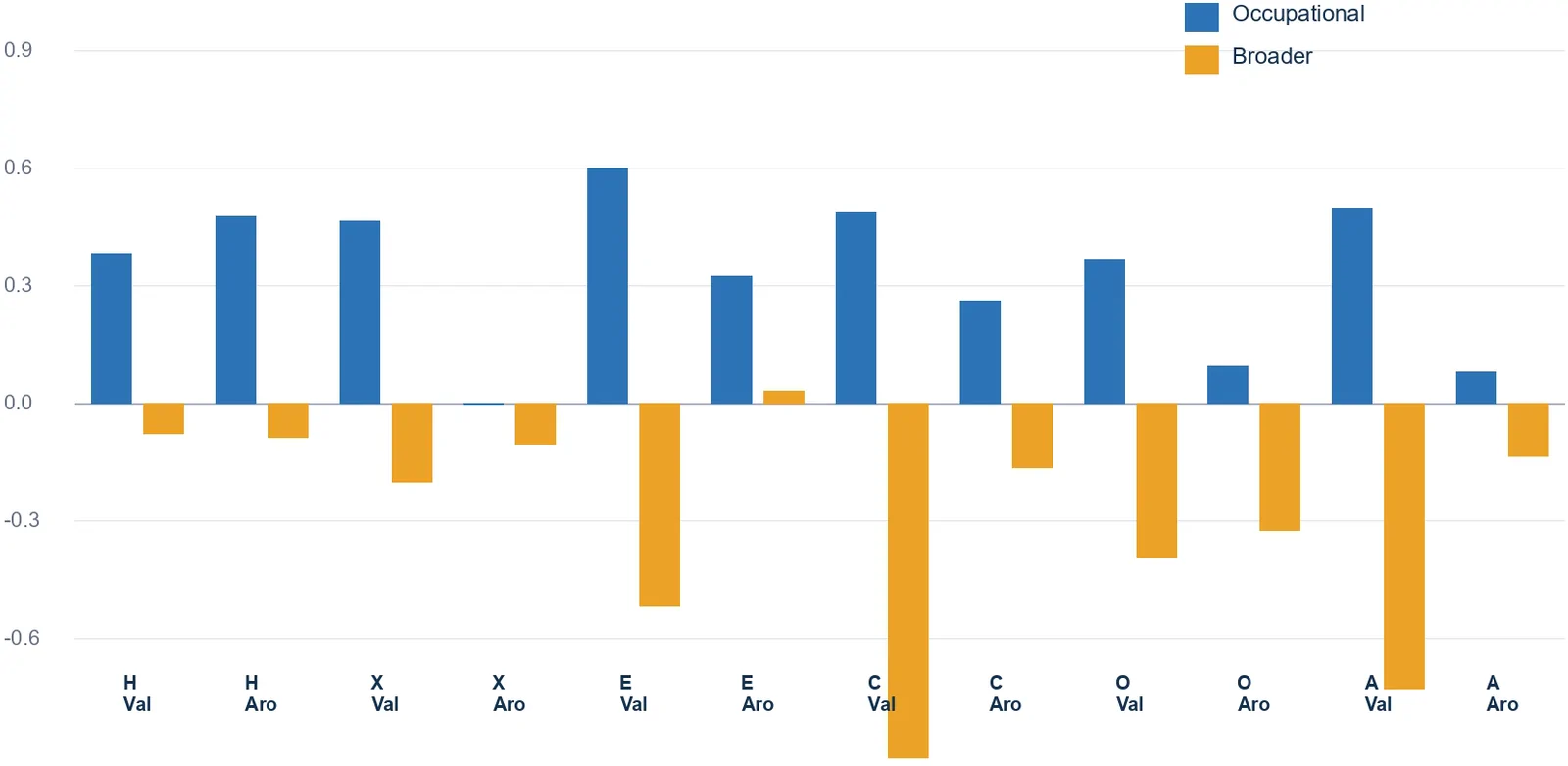
Observer reliability for valence and arousal ratings. ICC(1,k) values are shown separately by question and protocol; broader-protocol ratings were not stable enough for substantive correlational interpretation.

### 3.4 Same-observer personality, affect, and competence impressions

**Figure 4** shows strong associations among ratings made by the same observer pool. In the occupational protocol, all six focused personality-competence pairs survived correction: Extraversion with interacting/presenting ( r=0.71), Agreeableness with supporting/cooperating ( r=0.84), Openness with creating/conceptualizing ( r=0.75), Conscientiousness with organizing/executing ( r=0.62), Honesty-Humility with leading/deciding ( r=0.55), and Emotionality with adapting/coping ( r=−0.51; all q<0.001).

**Figure 4:**
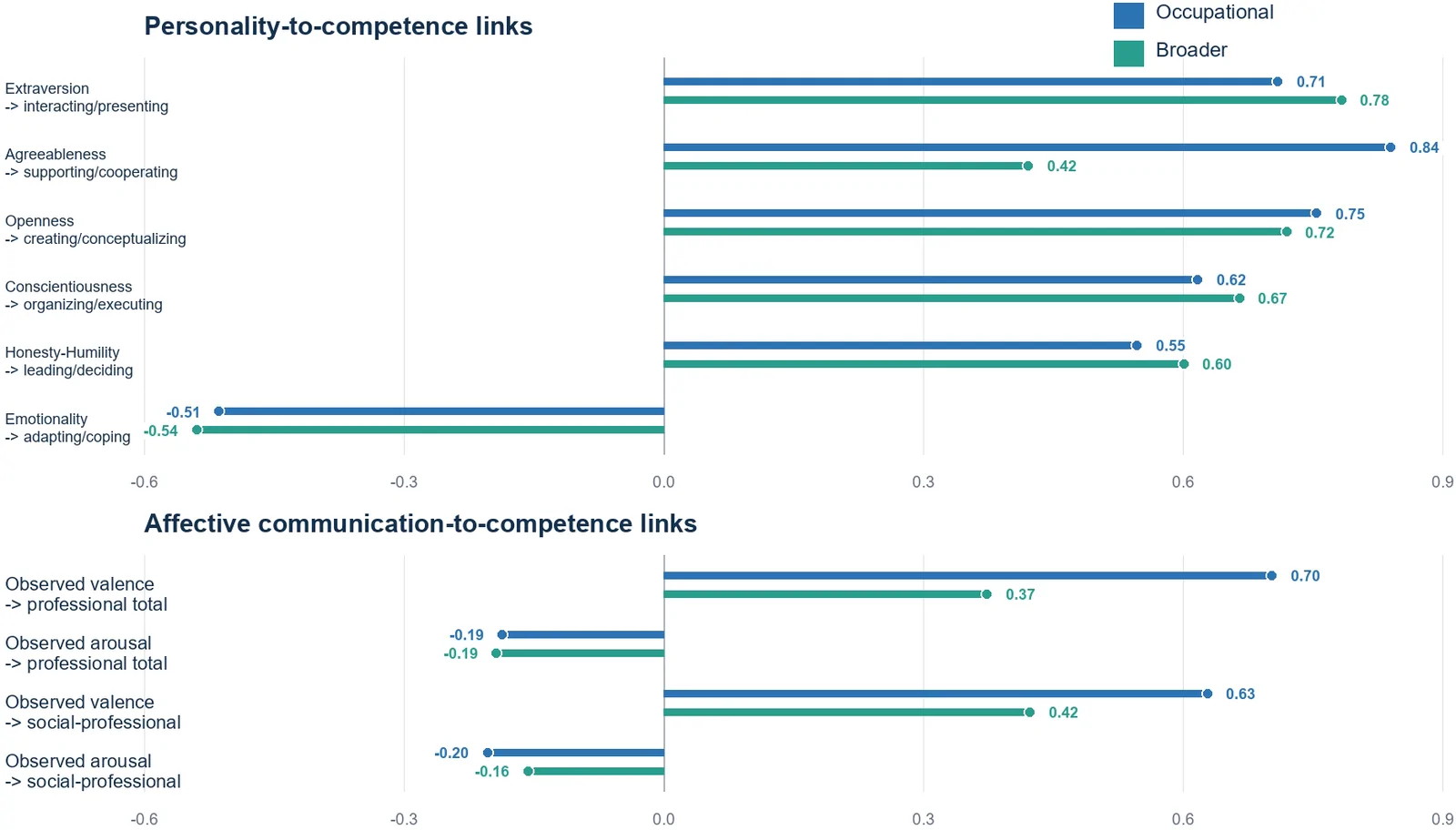
Focused domain-matched associations among personality expression, affective communication, and competence impressions rated by the same observer pool. Confidence intervals and adjusted q-values are reported in **Supplementary Table S1**.

The broader protocol showed a similar pattern: Extraversion with interacting/presenting ( r=0.78), Openness with creating/conceptualizing ( r=0.72), Conscientiousness with organizing/executing ( r=0.67), Honesty-Humility with leading/deciding ( r=0.60), Agreeableness with supporting/cooperating ( r=0.42), and Emotionality with adapting/coping ( r=−0.54) all survived correction ( q≤0.015). Fisher-transformed 95% confidence intervals are reported in **Supplementary Table S1**.

Rank-based sensitivity analyses preserved the direction and ordering of the largest associations, reducing concern that the pattern was solely a linear-model artifact. They do not address shared method variance, overlapping anchors, or halo effects.

Observed valence was associated with the professional total ( r=0.70, q<0.001) and social-professional composite ( r=0.63, q<0.001) in the occupational protocol. The corresponding broader-protocol estimates ( r=0.37 and r=0.42) did not survive correction ( q=0.069 and q=0.060) and are not substantively interpreted because broader affect reliability was poor. Observed arousal associations were small and did not survive correction in either protocol.

**Figure 5** displays the full exploratory matrices. Broad positive blocks were visible for several personality-expression dimensions, observed valence, and competence impressions. Because these cells share raters and were not the focused analysis family, they are used to visualize impression structure rather than to claim distinct trait-competence mechanisms.

**Figure 5:**
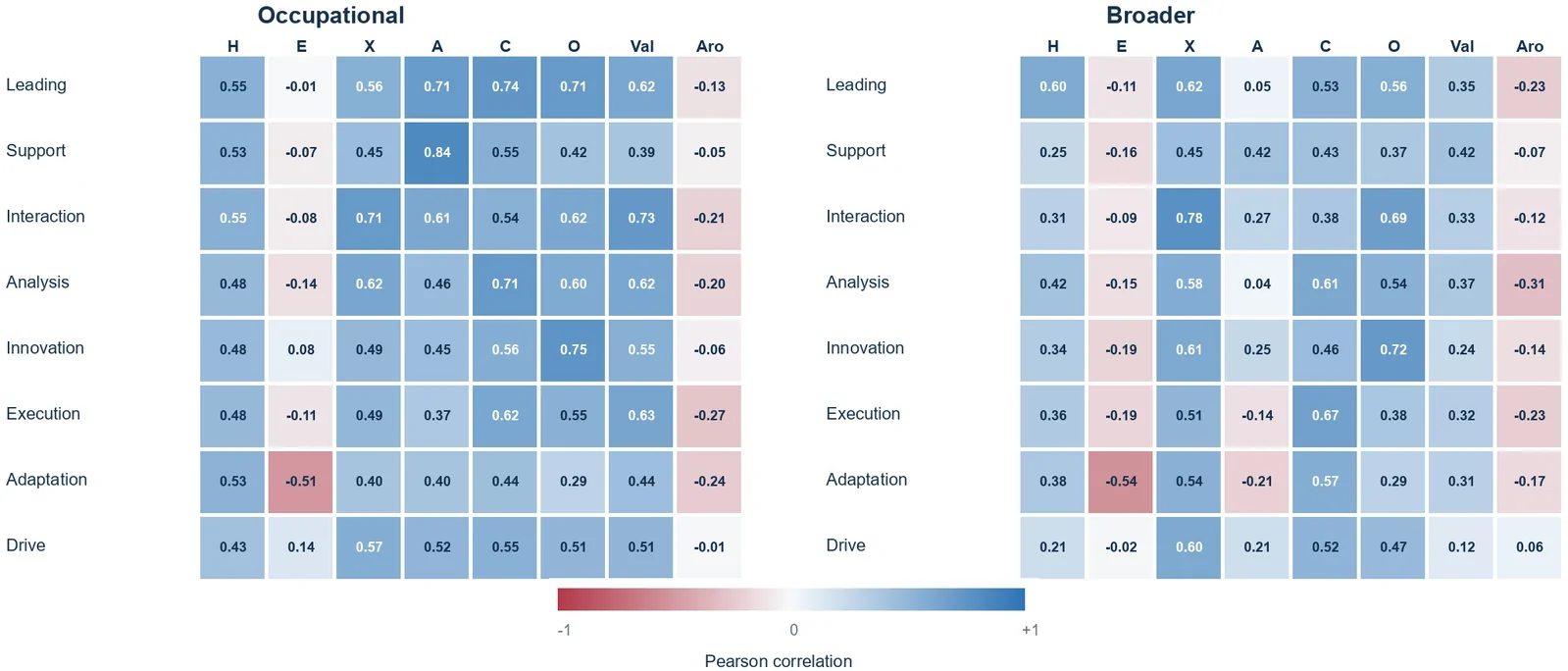
Exploratory correlation matrices among observer-rated personality expression, affective communication, and competence impressions. Individual cells are descriptive; focused inferential results appear in **Supplementary Table S1**.

The main descriptive exceptions were negative Emotionality-adapting/coping associations and near-zero or negative arousal associations. These patterns characterize how the observers organized their ratings. They do not show that emotionality or arousal predicts independently assessed performance.

Accordingly, the safest conclusion is observer-perceived coherence. The same four observers rated all three construct families, and some BARS descriptors can overlap conceptually. Shared method variance and halo effects can therefore inflate the associations. Independent rater teams or external performance criteria are required to test whether the pattern generalizes beyond this rating system.

## 4 Discussion

The recorded interview response is the common behavioral event connecting the study’s measurement layers. Observer reliability indicates whether raters extracted sufficiently consistent impressions from that event; self-observer associations indicate whether trait self-reports or experienced affect corresponded to what was expressed and perceived; and same-observer associations indicate how personality and affect impressions cohered with competence impressions. Interpreted together, the results characterize impression formation in a standardized, selection-like video-interview task rather than validate a hiring, performance, or clinical instrument.

### 4.1 Interpreting differences between the two protocols

The paired data separated relatively stable trait self-reports from more variable affective reports. Five HEXACO factors had small mean differences and all six retained moderate-to-high rank-order correlations. Agreeableness was lower in the broader protocol despite a high cross-protocol correlation, suggesting a mean-level shift in endorsement rather than wholesale reordering of participants. Global valence and selected question-level affect ratings differed more clearly.

A plausible explanation is that occupational questions imposed stronger evaluative or self-presentational demands, whereas broader questions allowed more positive personal material. The design cannot distinguish that explanation from topic, wording, emotional salience, or cognitive-demand differences. Accordingly, we describe the estimates as protocol differences and do not attribute them to occupational framing alone.

The secondary question-level results reinforce this caution. Differences were concentrated in the paired Honesty-Humility and Emotionality questions rather than distributed uniformly. Those pairs also differed noticeably in moral framing, responsibility, emotional reflection, and social support content. A future factorial design must hold question content constant while manipulating framing to estimate a contextual effect.

Thus, RQ1 is answered descriptively: personality self-reports were largely similar, whereas valence and selected question-level affect reports differed between the available protocols.

### 4.2 Trait self-reports and situation-specific expression

RQ2 compared measures that are related but not parallel. HEXACO self-reports summarize typical traits across many situations, whereas observers judged a narrow sample of behavior elicited by six questions. Occupational Extraversion and Agreeableness showed the clearest multiplicity-adjusted correspondence, consistent with the visibility of social activation and cooperative stance in brief interactions (16, 17). The other estimates do not demonstrate that internal characteristics failed to become socially interpretable. They can reflect limited behavioral sampling, different reference frames, and imperfect reliability (18).

No broader-protocol personality correlation survived correction. This difference could reflect weaker behavioral constraints, but it could also reflect the smaller sample and different question content. The study was not designed to separate these explanations. The affect results require an even stricter boundary. Occupational global valence showed modest correspondence, but occupational question-level affect reliability was uneven and broader-protocol reliability was extremely poor. Under these conditions, weak correlations cannot distinguish a true self-observer discrepancy from measurement error. We therefore avoid interpreting broader affect ratings as evidence of emotional unreadability or interpersonal misunderstanding (38).

Several non-exclusive measurement explanations are plausible. The broader questions invited heterogeneous autobiographical narratives and may have supplied less comparable affective cues across participants. Observers may also have applied different thresholds to vocal, facial, and verbal signals. Limited between-participant variation relative to rater noise would further depress the ICC estimates. The records document personality-rater calibration but no separate affect-coding curriculum or pre-coding reliability criterion. These features cannot be isolated as causes of the low ICCs, which should not be attributed to participants’ intrinsic emotional unreadability.

Accordingly, RQ2 identifies selective cross-method correspondence in the occupational protocol and substantial measurement constraints, rather than a general index of interpersonal readability.

### 4.3 Coherence within the observer-rating method

All six theory-guided personality-competence pairs survived multiplicity correction in both protocols. The pattern is consistent with internally coherent observer impressions: participants rated as more expressive on a HEXACO domain also tended to receive the corresponding competence rating. It does not establish a relationship between independently assessed personality and actual competence.

Occupational observed valence was also associated with the two competence composites after correction, whereas observed arousal was not. The broader valence associations did not survive correction and were based on unreliable affect ratings. We therefore do not infer that positive affect independently improves competence. A general favorable-impression halo could produce the same pattern.

Shared rater identity is central rather than peripheral to this interpretation. The same observers viewed the same responses and applied BARS that can contain overlapping behavioral cues. Future studies should assign independent rater teams to personality, affect, and competence or use an external performance criterion. Only such a design can test whether the present coherence reflects distinct constructs rather than shared method variance or halo effects.

Thus, RQ3 describes robust coherence within a common observer method, with the occupational affect pattern as a more tentative component.

### 4.4 Potential applications in young-adult mental-health research

One potential application is to use simulated interviews alongside validated symptom and functioning measures in studies of social-evaluative anxiety and emotional difficulties. The same task could inform research on adjustment during the transition from education to work. Observer ratings could then provide a behavioral perspective on how affect and interpersonal characteristics are expressed during evaluation, complementing rather than replacing participants’ accounts. The present findings identify reliability and self-observer correspondence as conditions that such research must address: poorly agreed affect ratings cannot support interpretable individual-level inferences. The current sample was non-clinical and did not assess psychiatric symptoms, risk, treatment, belonging, loneliness, or support. Future clinical or prevention studies should test whether independently coded ratings relate to symptoms or functioning and add information beyond self-report before considering assessment or screening applications.

## 5 Limitations

First, the samples were modest ( n=55, n=33, and n=30 paired), university-based, non-clinical, and restricted to emerging adults aged 18–23 years. The study was not preregistered, no *a priori* power analysis was conducted, and no prospective stopping rule was documented. The sensitivity analysis shows that the realized samples provided 80% power only for effects of at least $|d_{z}|=0.53$ in the paired comparison, |r|=0.37 in the occupational protocol, and |r|=0.47 in the broader protocol under an unadjusted two-sided α=0.05. Smaller effects can therefore have gone undetected, whereas isolated observed associations can be sample-specific and require replication in larger samples.

Second, the two protocols differed in item content as well as framing, so protocol differences cannot be attributed specifically to occupational versus general context. Third, trait self-reports and brief observer expression ratings have different reference frames and cannot be treated as parallel measures of the same construct.

Fourth, the same observer pool rated personality expression, affective communication, and competence, creating shared method variance and possible halo effects. The procedural records describe personality-rater preparation and calibration but do not document a separate affect/competence training curriculum, a numerical pre-coding reliability threshold, or formal blinding. These undocumented features cannot be evaluated retrospectively. Fifth, affect reliability was uneven in the occupational protocol and extremely poor in the broader protocol, limiting all observer-affect analyses. Finally, the study did not measure social connectedness, loneliness, belonging, support, psychiatric symptoms, self-harm risk, treatment response, or objective performance. The results are not a social-connectedness profile, a clinical screen, or a validated risk or competence marker.

## 6 Future works

Larger preregistered studies should use content-matched questions, justify sample size prospectively, and report confidence intervals and multiplicity control. Direct measures of belonging, loneliness, perceived support, and psychiatric symptoms would be required to test whether any behavioral or cross-method measure relates to psychosocial functioning. Longitudinal designs would be needed for prognostic claims.

Methodologically, independent rater teams should code personality expression, affective communication, and competence. Affect coders need explicit behavioral anchors, calibration sessions, and a pre-specified reliability gate before formal scoring. External performance criteria and more naturalistic peer or group interactions would test whether the observed coherence generalizes beyond one rating system and one task format.

Only after those measurement steps should intervention studies test whether changes in narrative organization, emotion regulation, or social behavior correspond to changes in validated psychosocial outcomes.

## 7 Conclusions

In emerging adults, HEXACO self-reports were largely similar across two non-equivalent interview protocols, whereas reported valence and selected question-level affect differed. Self-observer correspondence was selective, and affect interpretation was constrained by poor inter-rater reliability. Personality-expression and competence ratings made by the same observers were strongly coherent, but this coherence cannot establish independent competence. The study identifies measurement conditions for using simulated interviews in future young-adult mental-health research alongside validated symptom and functioning measures. It does not measure psychiatric vulnerability or establish screening utility.

## Data Availability

The raw data supporting the conclusions of this article will be made available by the authors, without undue reservation.
