# Supplementary material for: Affective and interpersonal expression in young adults during simulated interviews: implications for mental health research
Source: Front Psychiatry. 2026 Oct 28;17:1946199. doi: 10.3389/fpsyt.2026.1946199 (PMC13752034; doi:10.3389/fpsyt.2026.1946199)
Supplement: Supplementary file 1 [file DataSheet1.pdf]

Supplementary Table S1. Multiplicity-adjusted focused and secondary analyses

All tests are two-sided. Paired protocol differences use deterministic sign-flip permutation tests; correlations use deterministic Monte Carlo permutation tests. Benjamini-Hochberg q-values were calculated within each analysis family and protocol. Correlation intervals use Fisher's transformation; mean-difference intervals use the paired t distribution. Full correlation matrices in the main manuscript are exploratory and individual cells are not treated as inferential tests.

Tier	Family	Protocol	Measure	n	Estimate (95% CI)	p	q
focused	RQ1 focused personality	matched	Honesty-Humility	30	MD=-0.02 [-0.14, 0.11]; $d_z = -0.05$	0.838	0.838
focused	RQ1 focused personality	matched	Emotionality	30	MD=0.05 [-0.10, 0.21]; $d_z = 0.13$	0.510	0.838
focused	RQ1 focused personality	matched	Extraversion	30	MD=-0.04 [-0.17, 0.09]; $d_z = -0.10$	0.609	0.838
focused	RQ1 focused personality	matched	Agreeableness	30	MD=-0.23 [-0.35, -0.12]; $d_z = -0.76$	< 0.001	0.004
focused	RQ1 focused personality	matched	Conscientiousness	30	MD=0.02 [-0.10, 0.15]; $d_z = 0.07$	0.743	0.838
focused	RQ1 focused personality	matched	Openness	30	MD=0.06 [-0.10, 0.21]; $d_z = 0.14$	0.486	0.838
focused	RQ1 focused global affect	matched	Global valence	30	MD=0.30 [0.15, 0.45]; $d_z = 0.76$	< 0.001	< 0.001
focused	RQ1 focused global affect	matched	Global arousal	30	MD=-0.18 [-0.38, 0.02]; $d_z = -0.33$	0.088	0.088
secondary	RQ1 secondary question affect	matched	Honesty-Humility question: valence	30	MD=0.63 [0.24, 1.03]; $d_z = 0.59$	0.004	0.029
secondary	RQ1 secondary question affect	matched	Honesty-Humility question: arousal	30	MD=-0.57 [-0.96, -0.18]; $d_z = -0.54$	0.010	0.040
secondary	RQ1 secondary question affect	matched	Emotionality question: valence	30	MD=0.57 [0.22, 0.92]; $d_z = 0.61$	0.005	0.029
secondary	RQ1 secondary question affect	matched	Emotionality question: arousal	30	MD=-0.30 [-0.64, 0.04]; $d_z = -0.33$	0.124	0.335
secondary	RQ1 secondary question affect	matched	Extraversion question: valence	30	MD=0.23 [-0.04, 0.51]; $d_z = 0.32$	0.149	0.335
secondary	RQ1 secondary question affect	matched	Extraversion question: arousal	30	MD=-0.30 [-0.68, 0.08]; $d_z = -0.29$	0.167	0.335
secondary	RQ1 secondary question affect	matched	Agreeableness question: valence	30	MD=0.03 [-0.28, 0.35]; $d_z = 0.04$	1.000	1.000
secondary	RQ1 secondary question affect	matched	Agreeableness question: arousal	30	MD=0.10 [-0.22, 0.42]; $d_z = 0.12$	0.661	0.806
secondary	RQ1 secondary question affect	matched	Conscientiousness question: valence	30	MD=0.23 [-0.10, 0.57]; $d_z = 0.26$	0.231	0.396
secondary	RQ1 secondary question affect	matched	Conscientiousness question: arousal	30	MD=-0.10 [-0.44, 0.24]; $d_z = -0.11$	0.697	0.806
secondary	RQ1 secondary question affect	matched	Openness question: valence	30	MD=0.10 [-0.15, 0.35]; $d_z = 0.15$	0.591	0.806
secondary	RQ1 secondary question affect	matched	Openness question: arousal	30	MD=0.10 [-0.30, 0.50]; $d_z = 0.09$	0.739	0.806

Supplementary Table S1 (continued). Occupational protocol

Tier	Family	Protocol	Measure	n	Estimate (95% CI)	p	q
focused	RQ2 focused personality	occupational	Honesty-Humility	55	$r = 0.05$ [-0.22, 0.31]	0.713	0.713
focused	RQ2 focused personality	occupational	Emotionality	55	$r = 0.20$ [-0.07, 0.44]	0.147	0.212
focused	RQ2 focused personality	occupational	Extraversion	55	$r = 0.41$ [0.16, 0.61]	0.002	0.010
focused	RQ2 focused personality	occupational	Agreeableness	55	$r = 0.33$ [0.08, 0.55]	0.011	0.033
focused	RQ2 focused personality	occupational	Conscientiousness	55	$r = 0.19$ [-0.08, 0.44]	0.163	0.212
focused	RQ2 focused personality	occupational	Openness	55	$r = 0.19$ [-0.08, 0.43]	0.177	0.212
focused	RQ2 focused global affect	occupational	Global valence	55	$r = 0.32$ [0.06, 0.54]	0.019	0.038
focused	RQ2 focused global affect	occupational	Global arousal	55	$r = 0.18$ [-0.09, 0.42]	0.194	0.194
secondary	RQ2 secondary question affect	occupational	Honesty-Humility question: valence	55	$r = 0.25$ [-0.02, 0.48]	0.067	0.266
secondary	RQ2 secondary question affect	occupational	Honesty-Humility question: arousal	55	$r = 0.13$ [-0.14, 0.38]	0.350	0.599
secondary	RQ2 secondary question affect	occupational	Emotionality question: valence	55	$r = 0.23$ [-0.04, 0.47]	0.100	0.298
secondary	RQ2 secondary question affect	occupational	Emotionality question: arousal	55	$r = 0.29$ [0.03, 0.52]	0.032	0.194
secondary	RQ2 secondary question affect	occupational	Extraversion question: valence	55	$r = 0.30$ [0.04, 0.52]	0.026	0.194
secondary	RQ2 secondary question affect	occupational	Extraversion question: arousal	55	$r = 0.07$ [-0.20, 0.33]	0.632	0.729
secondary	RQ2 secondary question affect	occupational	Agreeableness question: valence	55	$r = 0.20$ [-0.07, 0.44]	0.144	0.345
secondary	RQ2 secondary question affect	occupational	Agreeableness question: arousal	55	$r = 0.10$ [-0.17, 0.36]	0.471	0.707
secondary	RQ2 secondary question affect	occupational	Conscientiousness question: valence	55	$r = 0.05$ [-0.22, 0.31]	0.716	0.729
secondary	RQ2 secondary question affect	occupational	Conscientiousness question: arousal	55	$r = 0.05$ [-0.22, 0.31]	0.729	0.729
secondary	RQ2 secondary question affect	occupational	Openness question: valence	55	$r = 0.14$ [-0.13, 0.39]	0.333	0.599
secondary	RQ2 secondary question affect	occupational	Openness question: arousal	55	$r = 0.08$ [-0.19, 0.33]	0.600	0.729
focused	RQ3 focused domain-matched	occupational	Honesty-Humility - leading/deciding	55	$r = 0.55$ [0.33, 0.71]	< 0.001	< 0.001
focused	RQ3 focused domain-matched	occupational	Emotionality - adapting/coping	55	$r = -0.51$ [-0.69, -0.29]	< 0.001	< 0.001
focused	RQ3 focused domain-matched	occupational	Extraversion - interacting/presenting	55	$r = 0.71$ [0.55, 0.82]	< 0.001	< 0.001
focused	RQ3 focused domain-matched	occupational	Agreeableness - supporting/cooperating	55	$r = 0.84$ [0.74, 0.90]	< 0.001	< 0.001
focused	RQ3 focused domain-matched	occupational	Conscientiousness - organizing/executing	55	$r = 0.62$ [0.42, 0.76]	< 0.001	< 0.001
focused	RQ3 focused domain-matched	occupational	Openness - creating/conceptualizing	55	$r = 0.75$ [0.61, 0.85]	< 0.001	< 0.001
focused	RQ3 focused global affect	occupational	Observed valence - professional total	55	$r = 0.70$ [0.54, 0.82]	< 0.001	< 0.001
focused	RQ3 focused global affect	occupational	Observed valence - social professional	55	$r = 0.63$ [0.44, 0.77]	< 0.001	< 0.001
focused	RQ3 focused global affect	occupational	Observed arousal - professional total	55	$r = -0.19$ [-0.43, 0.08]	0.176	0.176
focused	RQ3 focused global affect	occupational	Observed arousal - social professional	55	$r = -0.20$ [-0.44, 0.07]	0.137	0.176

Supplementary Table S1 (continued). Broader protocol

Tier	Family	Protocol	Measure	n	Estimate (95% CI)	p	q
focused	RQ2 focused personality	broader	Honesty-Humility	33	$r = 0.17$ [-0.18, 0.49]	0.342	0.657
focused	RQ2 focused personality	broader	Emotionality	33	$r = 0.12$ [-0.24, 0.44]	0.512	0.657
focused	RQ2 focused personality	broader	Extraversion	33	$r = 0.34$ [0.00, 0.61]	0.050	0.301
focused	RQ2 focused personality	broader	Agreeableness	33	$r = -0.08$ [-0.41, 0.27]	0.657	0.657
focused	RQ2 focused personality	broader	Conscientiousness	33	$r = 0.13$ [-0.22, 0.46]	0.468	0.657
focused	RQ2 focused personality	broader	Openness	33	$r = 0.08$ [-0.27, 0.41]	0.654	0.657
focused	RQ2 focused global affect	broader	Global valence	33	$r = 0.13$ [-0.23, 0.45]	0.498	0.637
focused	RQ2 focused global affect	broader	Global arousal	33	$r = 0.09$ [-0.26, 0.42]	0.637	0.637
secondary	RQ2 secondary question affect	broader	Honesty-Humility question: valence	33	$r = 0.18$ [-0.18, 0.49]	0.331	0.864
secondary	RQ2 secondary question affect	broader	Honesty-Humility question: arousal	33	$r = 0.03$ [-0.31, 0.37]	0.859	1.000
secondary	RQ2 secondary question affect	broader	Emotionality question: valence	33	$r = -0.00$ [-0.34, 0.34]	1.000	1.000
secondary	RQ2 secondary question affect	broader	Emotionality question: arousal	33	$r = 0.17$ [-0.18, 0.49]	0.345	0.864
secondary	RQ2 secondary question affect	broader	Extraversion question: valence	33	$r = -0.05$ [-0.38, 0.30]	0.808	1.000
secondary	RQ2 secondary question affect	broader	Extraversion question: arousal	33	$r = 0.25$ [-0.10, 0.55]	0.166	0.864
secondary	RQ2 secondary question affect	broader	Agreeableness question: valence	33	$r = 0.05$ [-0.30, 0.39]	0.846	1.000
secondary	RQ2 secondary question affect	broader	Agreeableness question: arousal	33	$r = -0.20$ [-0.51, 0.15]	0.286	0.864
secondary	RQ2 secondary question affect	broader	Conscientiousness question: valence	33	$r = 0.08$ [-0.27, 0.41]	0.648	1.000
secondary	RQ2 secondary question affect	broader	Conscientiousness question: arousal	33	$r = 0.07$ [-0.28, 0.41]	0.713	1.000
secondary	RQ2 secondary question affect	broader	Openness question: valence	33	$r = 0.01$ [-0.33, 0.35]	0.952	1.000
secondary	RQ2 secondary question affect	broader	Openness question: arousal	33	$r = 0.17$ [-0.19, 0.48]	0.360	0.864
focused	RQ3 focused domain-matched	broader	Honesty-Humility - leading/deciding	33	$r = 0.60$ [0.33, 0.78]	< 0.001	< 0.001
focused	RQ3 focused domain-matched	broader	Emotionality - adapting/coping	33	$r = -0.54$ [-0.74, -0.24]	0.001	0.001
focused	RQ3 focused domain-matched	broader	Extraversion - interacting/presenting	33	$r = 0.78$ [0.60, 0.89]	< 0.001	< 0.001
focused	RQ3 focused domain-matched	broader	Agreeableness - supporting/cooperating	33	$r = 0.42$ [0.09, 0.67]	0.015	0.015
focused	RQ3 focused domain-matched	broader	Conscientiousness - organizing/executing	33	$r = 0.67$ [0.42, 0.82]	< 0.001	< 0.001
focused	RQ3 focused domain-matched	broader	Openness - creating/conceptualizing	33	$r = 0.72$ [0.50, 0.85]	< 0.001	< 0.001
focused	RQ3 focused global affect	broader	Observed valence - professional total	33	$r = 0.37$ [0.03, 0.64]	0.035	0.069
focused	RQ3 focused global affect	broader	Observed valence - social professional	33	$r = 0.42$ [0.09, 0.67]	0.015	0.060
focused	RQ3 focused global affect	broader	Observed arousal - professional total	33	$r = -0.19$ [-0.50, 0.16]	0.282	0.376
focused	RQ3 focused global affect	broader	Observed arousal - social professional	33	$r = -0.16$ [-0.47, 0.20]	0.392	0.392

Supplementary Table S2. Observer reliability estimates

ICC(1,k) is the one-way random-effects average-measures intraclass correlation across four observers. Negative values indicate that between-participant variance was not greater than residual rater variance under the model; they do not indicate inverse agreement.

Protocol	Construct	Measure	ICC(1,k)
occupational	personality expression	Honesty-Humility	0.57
occupational	personality expression	Emotionality	0.27
occupational	personality expression	Extraversion	0.41
occupational	personality expression	Agreeableness	0.65
occupational	personality expression	Conscientiousness	0.53
occupational	personality expression	Openness	0.37
occupational	competence impression	leading deciding	0.36
occupational	competence impression	supporting cooperating	0.38
occupational	competence impression	interacting presenting	0.58
occupational	competence impression	analyzing interpreting	0.64
occupational	competence impression	creating conceptualizing	0.40
occupational	competence impression	organizing executing	0.35
occupational	competence impression	adapting coping	0.47
occupational	competence impression	enterprising performing	0.46
occupational	affective communication	Honesty-Humility question: valence	0.38
occupational	affective communication	Honesty-Humility question: arousal	0.47
occupational	affective communication	Emotionality question: valence	0.60
occupational	affective communication	Emotionality question: arousal	0.32
occupational	affective communication	Extraversion question: valence	0.46
occupational	affective communication	Extraversion question: arousal	-0.00
occupational	affective communication	Agreeableness question: valence	0.50
occupational	affective communication	Agreeableness question: arousal	0.08
occupational	affective communication	Conscientiousness question: valence	0.49
occupational	affective communication	Conscientiousness question: arousal	0.26
occupational	affective communication	Openness question: valence	0.37
occupational	affective communication	Openness question: arousal	0.09

Supplementary Table S2 (continued). Broader protocol

Protocol	Construct	Measure	ICC(1,k)
broader	personality expression	Honesty-Humility	0.48
broader	personality expression	Emotionality	0.33
broader	personality expression	Extraversion	0.58
broader	personality expression	Agreeableness	0.48
broader	personality expression	Conscientiousness	0.40
broader	personality expression	Openness	0.37
broader	competence impression	leading deciding	0.35
broader	competence impression	supporting cooperating	0.22
broader	competence impression	interacting presenting	0.50
broader	competence impression	analyzing interpreting	0.73
broader	competence impression	creating conceptualizing	0.40
broader	competence impression	organizing executing	0.22
broader	competence impression	adapting coping	0.51
broader	competence impression	enterprising performing	0.68
broader	affective communication	Honesty-Humility question: valence	-0.08
broader	affective communication	Honesty-Humility question: arousal	-0.09
broader	affective communication	Emotionality question: valence	-0.52
broader	affective communication	Emotionality question: arousal	0.03
broader	affective communication	Extraversion question: valence	-0.20
broader	affective communication	Extraversion question: arousal	-0.11
broader	affective communication	Agreeableness question: valence	-0.73
broader	affective communication	Agreeableness question: arousal	-0.14
broader	affective communication	Conscientiousness question: valence	-0.91
broader	affective communication	Conscientiousness question: arousal	-0.16
broader	affective communication	Openness question: valence	-0.40
broader	affective communication	Openness question: arousal	-0.33
